# Supplementary figures and images for: Treatment with sodium (S)-2-hydroxyglutarate prevents liver injury in an ischemia-reperfusion model in female Wistar rats
Source: PeerJ. 2021 Nov 12;9:e12426. doi: 10.7717/peerj.12426 (PMC8592047; doi:10.7717/peerj.12426)

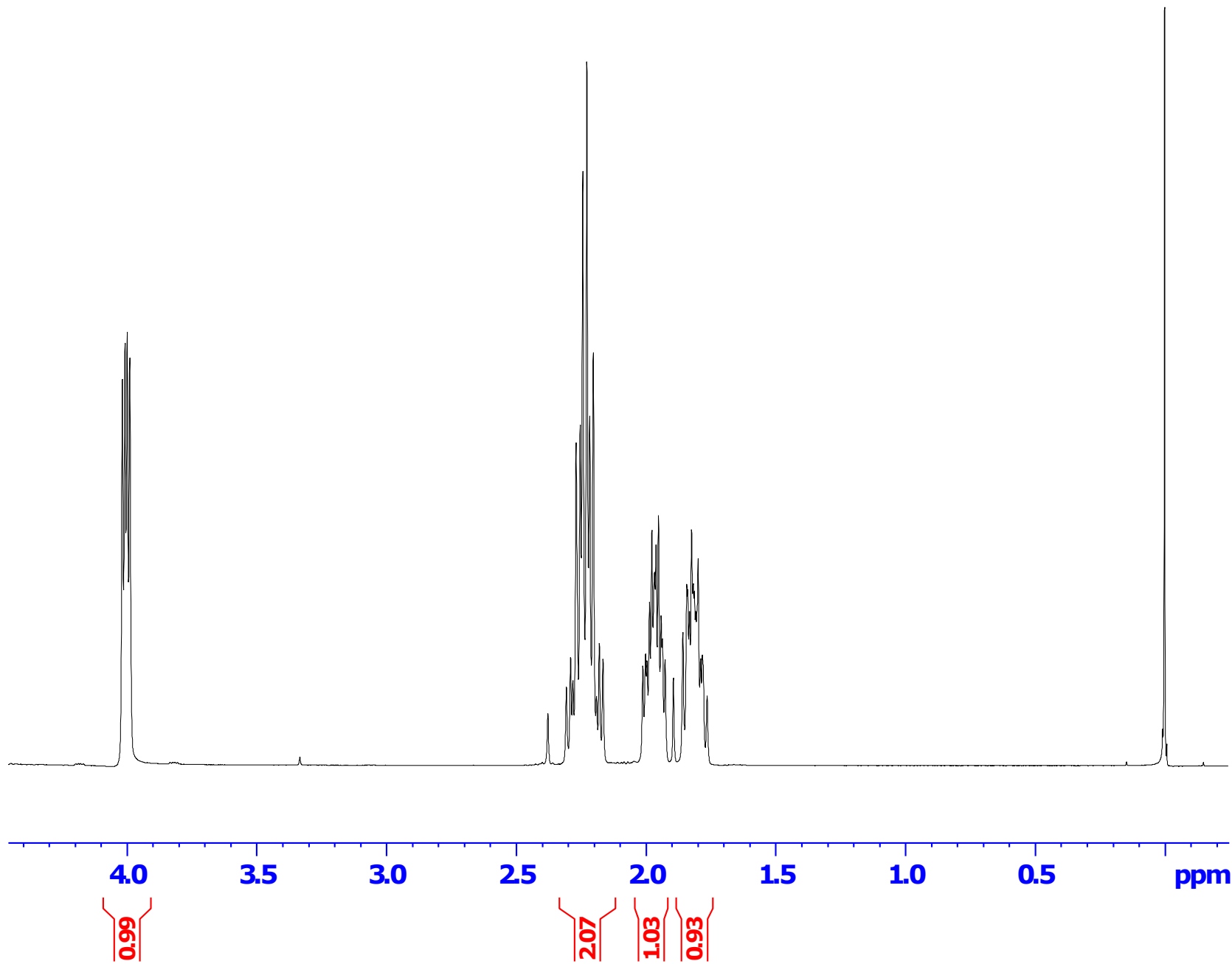

Supplement: Supplemental Information 1 [file peerj-09-12426-s001.pdf]

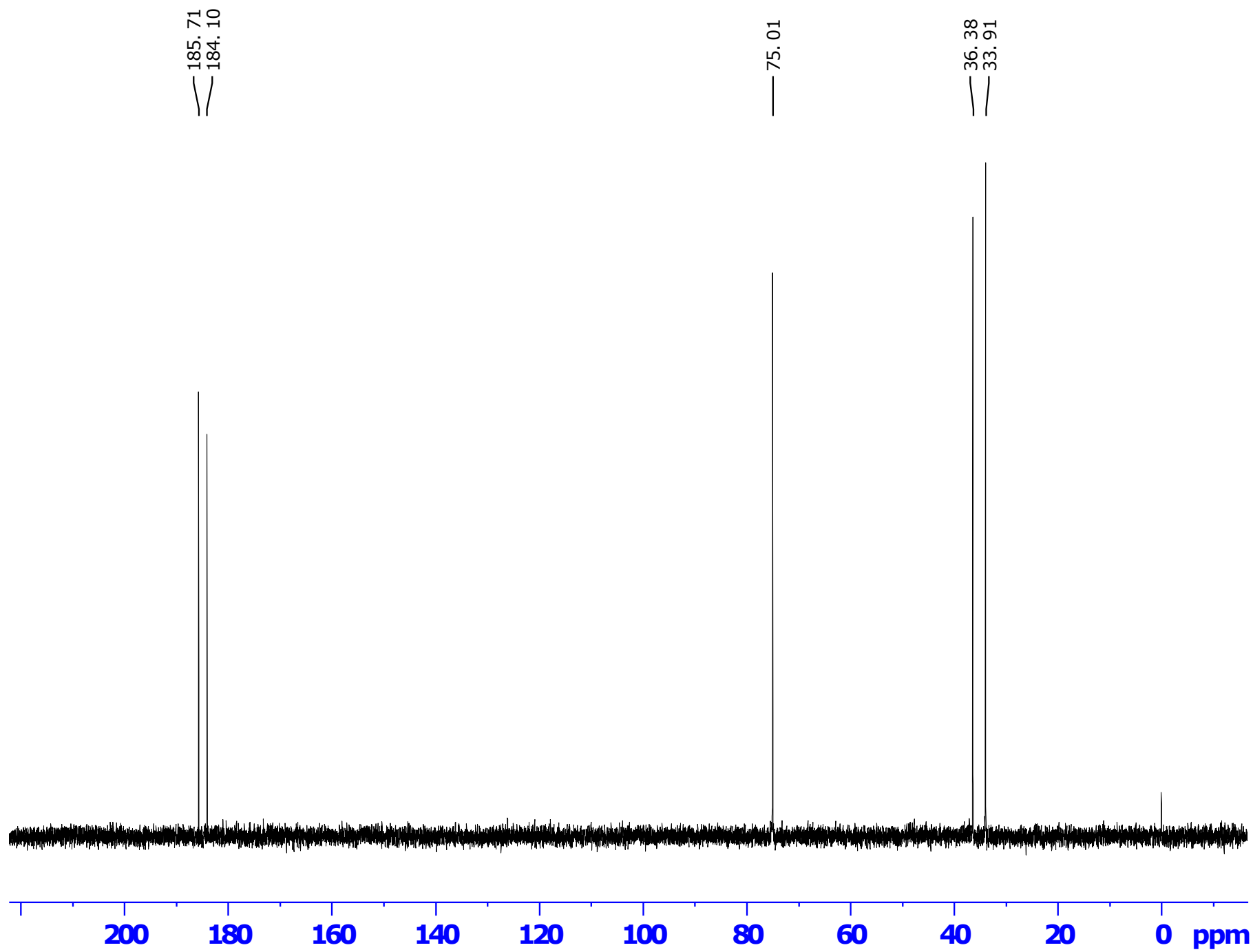

Supplement: Supplemental Information 2 [file peerj-09-12426-s002.pdf]

**A**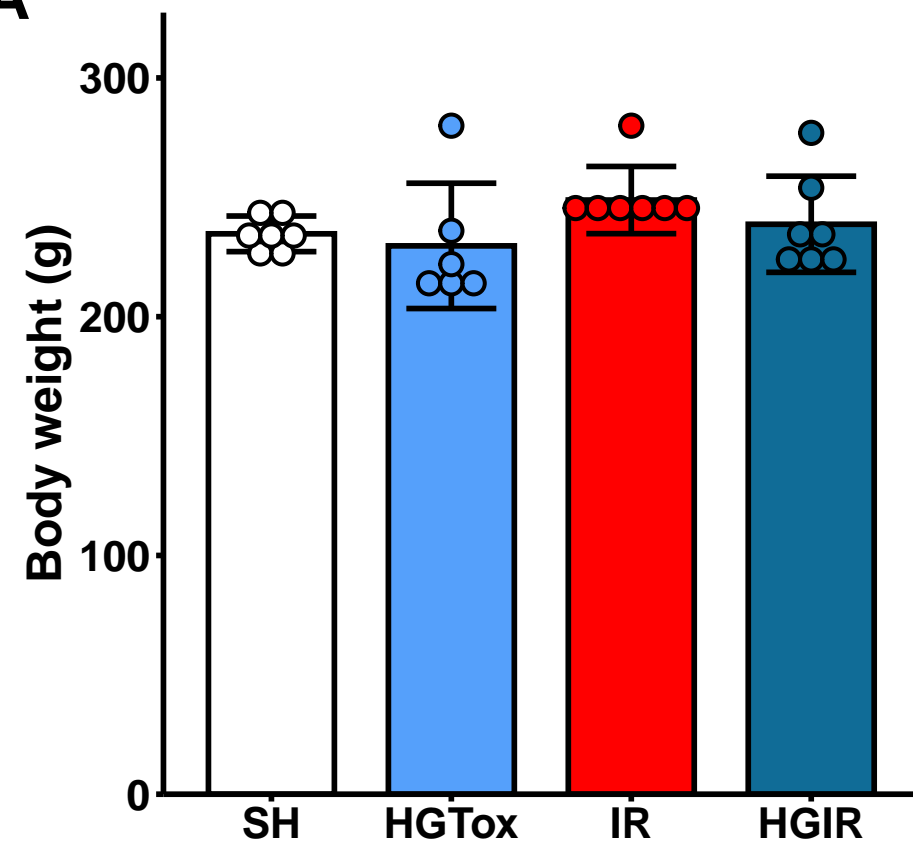**B**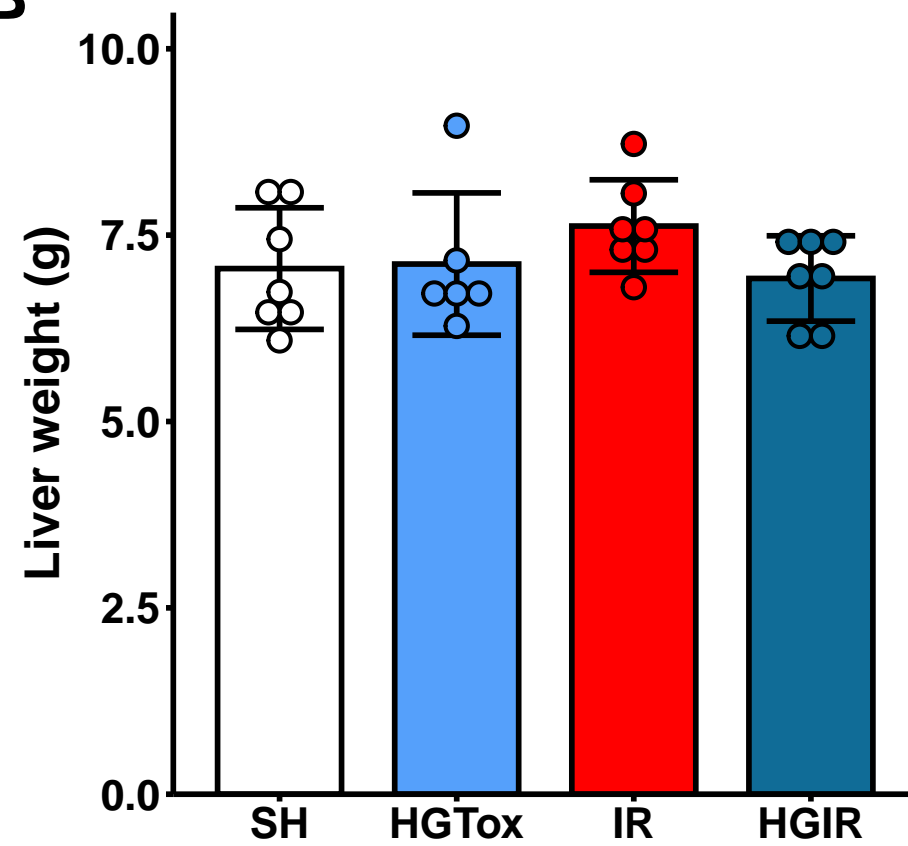**C**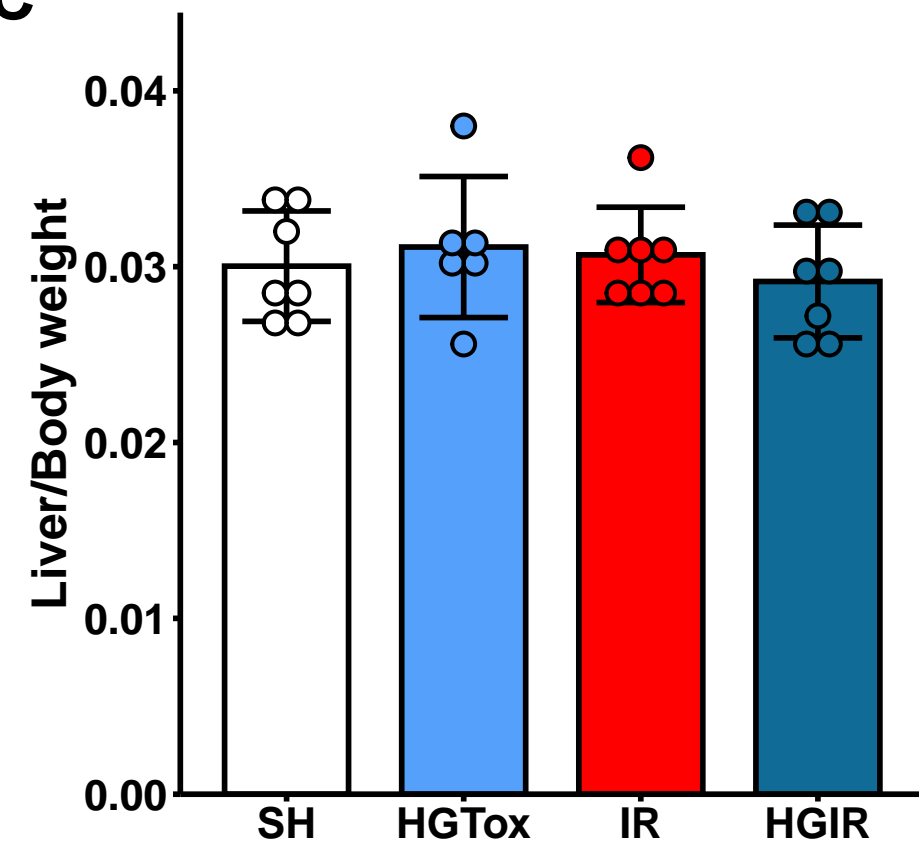

Supplement: Supplemental Information 3 — a) Body weight, b) liver weight, c) liver to body weight ratio. [file peerj-09-12426-s003.pdf]
